# Cutting the Brakes on Ras—Cytoplasmic GAPs as Targets of Inactivation in Cancer

**DOI:** 10.3390/cancers12103066

**Published:** 2020-10-21

**Authors:** Arianna Bellazzo, Licio Collavin

**Affiliations:** Department of Life Sciences, University of Trieste, Via L. Giorgieri 1, 34127 Trieste, Italy; abellazzo@units.it

**Keywords:** cell signaling, GTPase-Activating Proteins, tumor suppressor genes, signal transduction, mechanisms of transformation, RAS oncogene

## Abstract

**Simple Summary:**

GTPase-Activating Proteins (RasGAPs) are a group of structurally related proteins with a fundamental role in controlling the activity of Ras in normal and cancer cells. In particular, loss of function of RasGAPs may contribute to aberrant Ras activation in cancer. Here we review the multiple molecular mechanisms and factors that are involved in downregulating RasGAPs expression and functions in cancer. Additionally, we discuss how extracellular stimuli from the tumor microenvironment can control RasGAPs expression and activity in cancer cells and stromal cells, indirectly affecting Ras activation, with implications for cancer development and progression.

**Abstract:**

The Ras pathway is frequently deregulated in cancer, actively contributing to tumor development and progression. Oncogenic activation of the Ras pathway is commonly due to point mutation of one of the three Ras genes, which occurs in almost one third of human cancers. In the absence of Ras mutation, the pathway is frequently activated by alternative means, including the loss of function of Ras inhibitors. Among Ras inhibitors, the GTPase-Activating Proteins (RasGAPs) are major players, given their ability to modulate multiple cancer-related pathways. In fact, most RasGAPs also have a multi-domain structure that allows them to act as scaffold or adaptor proteins, affecting additional oncogenic cascades. In cancer cells, various mechanisms can cause the loss of function of Ras inhibitors; here, we review the available evidence of RasGAP inactivation in cancer, with a specific focus on the mechanisms. We also consider extracellular inputs that can affect RasGAP levels and functions, implicating that specific conditions in the tumor microenvironment can foster or counteract Ras signaling through negative or positive modulation of RasGAPs. A better understanding of these conditions might have relevant clinical repercussions, since treatments to restore or enhance the function of RasGAPs in cancer would help circumvent the intrinsic difficulty of directly targeting the Ras protein.

## 1. Introduction

Ras proteins (K-Ras, N-Ras, and H-Ras) are small monomeric GTPases that modulate cell fate by linking receptor activation to intracellular signaling, thereby controlling cell growth, survival, migration, and metabolism [1].

Ras signaling can be initiated by receptor tyrosine kinases (RTKs), G-protein coupled receptors (GPCRs), and integrin family members (reviewed in [2]). Once activated, Ras proteins trigger a number of pro-oncogenic signaling cascades, including mitogen-activated protein (MAP) kinases, the phosphoinositide 3-kinase/Akt (PI3K/Akt) axis, the NF-κB (nuclear factor kappa-light-chain-enhancer of activated B cells) transcription factor, and the mTOR (mammalian target of rapamycin) cytoplasmic regulator, supporting evasion of apoptosis, epithelial–mesenchymal transition (EMT), as well as metabolic and inflammatory cell reprogramming [2]. Ras proteins can also drive cytoskeletal reorganization and cell motility by stimulating the Rac-Rho and Rac-PAX networks [1,2].

In cancer cells, aberrant Ras activation establishes a complex oncogenic circuit that promotes tumor initiation, growth, and dissemination, to such an extent that some authors have proposed the concept of Ras-driven cancer [2]. In a large-scale analysis of the patterns of somatic alterations in cancer, the RTK-Ras axis turned out to be the canonical pathway with the highest median frequency of mutation [3]. Indeed, Ras proteins are frequently altered in tumors, with activating mutations identified in around one third of all human cancers [4,5]. Notably, in tumors without RAS mutation, multiple events can phenocopy Ras hyperactivation [6]. For instance, increased Ras pathway activity is detected in more that 50% of breast carcinomas, although the frequency of RAS mutation is much lower in this tumor type [4,7,8]. Similarly, evidence of activated Ras signaling is consistently found in liver cancer and myeloma, tumors that have a lower RAS mutation rate compared to other cancers [4,5,9,10].

Like most GTPases, Ras activity is controlled by activators and inhibitors; therefore, in the absence of RAS gene alterations, one important mechanism of Ras hyperactivation in cancer is the loss of function of Ras inhibitors. Here, we analyze one major class of Ras inhibitors: the GTPase-Activating Proteins (RasGAPs).

## 2. The Brakes: GAPs as Negative Regulators of Ras Activity

**Figure 1:**
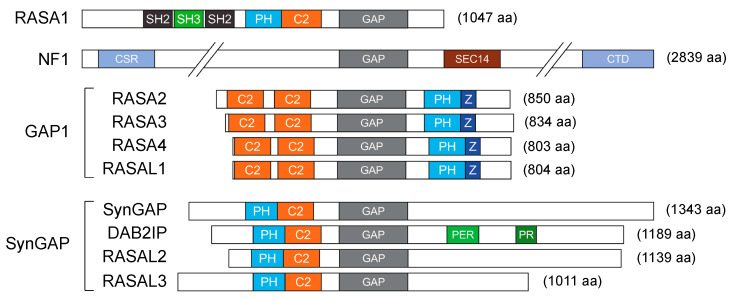
Domain organization of RasGAPs (Ras GTPase-Activating Proteins). In addition to the GAP domain, different RasGAP proteins are characterized by a specific array of functional domains, orchestrating their subcellular localization and regulation (SH2 = Src homology 2 domain; SH3 = Src homology 3 domain; PH = plekstrin homology domain; C2 = calcium-dependent phospholipid-binding motif; CSR = Cys/Ser-rich region; SEC14 = CRAL-TRIO lipid-binding domain; CTD = C-terminal domain; Z = Btk-type zinc finger motif; PER = period-like domain; PR = proline-rich region). Representative proteins are drawn based on UniProtKB entries (2020_05 release, https://www.uniprot.org/). Note that NF1 is not drawn to scale.

The duration of Ras activity is tightly controlled; the conversion from the active GTP-bound form to the inactive GDP-bound state, intrinsic in Ras proteins, is accelerated by members of the GAP family. Consequently, loss of function of RasGAPs leads to aberrant Ras activation and increases downstream oncogenic signaling. Currently, there are 14 recognized RasGAP genes in the human genome; they all contain a GAP domain, but share limited similarity outside this region [11,12]. A set of additional modules is common in RasGAPs, such as Ca^2+^-binding domains (C2) and phosphatidylinositol lipid-binding motifs (pleckstrin homology (PH)). The protein architecture and specific domains define six RasGAP subfamilies of shared structure and function (Figure 1; for a detailed description see [11,12]).

1. RASA1 (Ras p21 protein activator 1)/p120GAP was the first RasGAP to be identified. In early studies, RASA1 appeared to be essential for embryonic blood vessel development [13]; subsequently, a germline mutation in RASA1 was associated with vascular malformations [14]. Although RASA1 can mitigate Ras activity, its role in cancer has remained unclear for a long time; recent works described RASA1 inhibition by non-coding RNA in multiple aggressive tumors, supportive of a tumor-suppressive activity [15,16,17,18,19].
2. Neurofibromin (NF1) is perhaps the most extensively studied RasGAP. Germline mutations of NF1 are associated with the familiar disorder Neurofibromatosis Type 1. Germline NF1 mutations cause tumors along the nervous system, including neurofibromas and malignant peripheral nerve sheath tumors (MPNSTs). NF1 patients are also predisposed to develop gliomas, melanoma, and myeloid leukemia [20]. NF1 is also somatically altered in multiple sporadic tumors, including skin, lung, breast, and ovarian cancers [21], where its tumor-suppressive function seems to be linked primarily to Ras inhibition [11].
3. The GAP1 family includes RASA2 (Ras p21 protein activator 2)/GAP1^m^, RASA3 (Ras p21 protein activator 3)/GAP^IP4BP^, RASA4 (Ras p21 protein activator 4)/CAPRI, and RASAL1 (Ras protein activator like 1). Members of this group share large significant sequence homology and act as dual GAPs, binding and inactivating Ras and Rap1 GTPases. All members of this group are clearly implicated in cancer. For example, the recurrent inactivation of RASA2 in melanoma favors constitutive activation of Ras signaling [22], and the frequent downregulation of RASA4 in myelomonocytic leukemia correlates with poor prognosis and higher risk of relapse after therapy [23]. Furthermore, the recurrent deletion of chromosome 13, which includes the RASA3 gene, in Burkitt and T-cell lymphomas and in acute myeloid leukemia (AML) suggests a potential role of RASA3 in these blood tumors [24]. Finally, RASAL1 displays MAPK- and PI3K-suppressing activities in multiple tumors, including thyroid cancer [25,26].
4. The SynGAP family includes SynGAP (synaptic Ras GTPase-Activating Protein 1), DAB2IP (Dab2 interacting protein), RASAL2 (Ras protein activator like 2), and RASAL3 (Ras protein activator like 3). The founding member SynGAP is expressed only in neuronal tissue; germline SynGAP mutations have been associated with intellectual disabilities and autism [27]. RASAL2 functions as a tumor suppressor in a broad range of human tumors, including lung, ovarian, breast, and bladder cancer; low RASAL2 expression often correlates with aberrant Ras-ERK activation and worst prognosis [28,29]. Curiously, RASAL2 has also been described as an oncogene, based on its ability to promote EMT and metastasis in several tumors, fostering YAP (Yes1 associated transcriptional regulator), Wnt/β-catenin, PI3K/Akt, and Rac1 signaling [28]. DAB2IP is a tumor suppressor whose expression and function are altered in multiple human malignancies, causing uncontrolled activation of multiple oncogenic pathways, including Ras, NF-κB, PI3K/Akt, and Wnt/β-catenin [30,31]. Much less studied, RASAL3 epigenetic silencing in fibroblasts was linked to reprogramming of the tumor stroma in prostate cancer patients failing androgen deprivation therapy [32].
5. The IQGAPs (IQ motif containing GTPase-Activating Protein) are cytoskeletal scaffold proteins with high sequence homology but variable tissue distribution. Despite the presence of a GAP domain, the IQGAPs are not Ras inhibitors, since the GAP domain lacks an arginine essential to assist Ras in GTP hydrolysis [11]. For this reason, they will not be considered here.
6. Plexins are transmembrane receptors that bind semaphorins and regulate cell migration and angiogenesis. Plexins display GAP activity to Ras and Rap (a sub-family of Ras homologs) so they have a dual specificity [33]. Plexins also bind and modulate cytosolic tyrosine kinases, serine/threonine kinases, and other adaptors and scaffolding proteins, orchestrating complex signaling networks. Functional alterations in plexins cause cardiovascular and neuronal disorders, but their role in cancer is unclear: loss of plexins, specially plexin B1, was correlated with aggressive tumors [34]. However, there is also evidence of an oncogenic role for plexins in multiple malignancies [35,36,37]. Given their role as receptors and their complex Ras-independent functions, plexins will not be considered in this review.

## 3. Mutation of RasGAPs in Cancer

There is some evidence that RasGAPs can be inactivated by mutation in cancer. The gene encoding NF1 is altered in hereditary Neurofibromatosis Type 1-associated tumors and in numerous sporadic cancers [21]. Specifically, NF1 is mutated in approximately 13% of cutaneous melanoma, together with inactivating the mutation of other tumor suppressor genes such as TP53, CDKN2A (cyclin dependent kinase inhibitor 2A), PTEN (phosphatase and tensin homolog), or RB1 (RB transcriptional corepressor 1) [38,39]. NF1 is also frequently mutated in desmoplastic melanoma, suggesting it may be a key factor in the biology of this tumor [40]. In addition, NF1 is mutated in a variety of other cancers, including lung adenocarcinoma, squamous cell carcinoma, breast and ovarian cancer, glioblastoma, and acute myeloid leukemia [41,42,43,44,45,46,47,48,49]. NF1 alterations can be various, but more than 80% of NF1 mutations result in almost complete absence of the transcript and protein, and drive oncogenic activation of Ras signaling, leading to highly proliferative tumors. Interestingly, in almost 10% of melanomas NF1 mutation co-occurs with RAS and BRAF alterations [21], suggesting a role for NF1 in controlling other pathways in addition to Ras-MAPK. For example, increased activation of the PI3K/Akt/mTOR axis was observed specifically in BRAF/NF1 double mutant tumors [50]. Recently, a systematic analysis of colorectal carcinoma samples revealed that NF1 is frequently altered in K-RAS G13-mutant tumors, but not in tumors with other K-RAS mutations. This observation led to the discovery that K-Ras G13D remains susceptible to NF1 GAP activity, so NF1 loss may provide a selective advantage to cancer cells with the K-RAS G13D mutation. It also implies that epidermal growth factor receptor (EGFR) inhibitors may be effective in K-RAS G13D mutant tumors that retain wild-type NF1 [51].

RASA1 mutations have been documented in hepatocellular carcinoma, breast cancer, and melanoma [52,53,54]. Recently, Hayashi et al. reported that RASA1 and NF1 are preferentially co-mutated in smoking-associated non-small cell lung carcinomas (NSCLCs) [55]. Intriguingly, the concurrent loss-of-function mutation of RASA1 and NF1 appears to be mutually exclusive with other mitogenic driver mutations (e.g., K-RAS or EGFR); the inactivation of both NF1 and RASA1 could therefore be sufficient to fully hyperactivate Ras signaling in the absence of mitogenic mutations [55]. Similarly, RASA2 gene alterations co-occur with NF1 mutation in melanoma, where RASA2 is mutated in about 5% of patients, leading to Ras signaling activation [22]. The RASAL1 gene was found to be mutated in thyroid cancer [25]. Finally, DAB2IP and RASAL2 mutations or deletions have been reported in different tumor types, including breast (both), gastrointestinal (DAB2IP), colorectal, lung, and ovarian cancer (RASAL2) [56,57,58,59,60,61]. Despite the above evidence, the overall frequency of mutation in GAP genes is relatively low in human tumors, in line with the observation that these proteins are frequently inactivated via other means (see below).

## 4. Transcriptional Repression of RasGAPs in Cancer

Numerous studies have described aberrant promoter methylation of RasGAPs in multiple cancers, with various de novo DNA methyl transferases involved. For instance, in hypoxic condition, transforming growth factor-beta (TGF-β) was shown to induce promoter methylation of RASAL1 through recruitment of DNA methyl transferase 1 (DNMT1) [62,63]. Under these conditions, RASAL1 repression causes Ras-dependent aberrant activation of fibroblasts, with increased proliferation and collagen type I production, leading to kidney fibrosis [62].

DAB2IP transcription is repressed by EZH2, the histone methyl transferase subunit of the Polycomb Repressor Complex (PCR) 2, which is often overexpressed in human tumors [64,65]. Various reports indicate EZH2-dependent hypermethylation of CpG islands in the DAB2IP promoter, correlated with reduced DAB2IP expression levels. Accordingly, EZH2 activity stimulates both Ras signaling and NF-κB activity, thereby promoting prostate tumor growth, EMT, and metastasis [66]. A recent report showed that EZH2-dependent DAB2IP repression induces a stem-like phenotype in ovarian cancer cells by unleashing WNT5B-dependent planar cell polarity signaling; this study also provides proof of concept that pharmacologic inhibition of EZH2 can restore DAB2IP expression and reduce stem features and aggressiveness of ovarian cancer cells [67].

Interestingly, aberrant methylation of RasGAPs can also occur in non-transformed cells within the tumor stroma. Hypermethylation of RASAL3 on exon 2 was observed in prostate cancer associated fibroblasts (CAFs), favoring Ras signaling and metabolic reprograming in CAFs and cancer cells, fostering their proliferation. Intriguingly, androgen deprivation therapy could promote this phenomenon, suggesting that androgen signaling prevents CpG methylation of the RASAL3 gene [32]. Additional reports of promoter methylation of various RasGAPs in cancer are summarized in Table 1.

We know surprisingly little about transcription factors that control RasGAP expression in various tissues. Nonetheless, there is at least one piece of evidence of cancer-related inactivation of a transcriptional regulator for a RasGAP gene. In fact, the homeodomain pituitary transcription factor PITX1 (paired like homeodomain 1) binds a consensus site in the RASAL1 promoter, stimulating its transcription [71]. Inactivation of PITX1 is common in multiple cancers, including prostate, bladder, and hepatocellular carcinoma, correlating with reduced RASAL1 expression and unconstrained Ras activation [9,71].

## 5. Post-Transcriptional Inhibition of RasGAPs in Cancer

**Figure 2:**
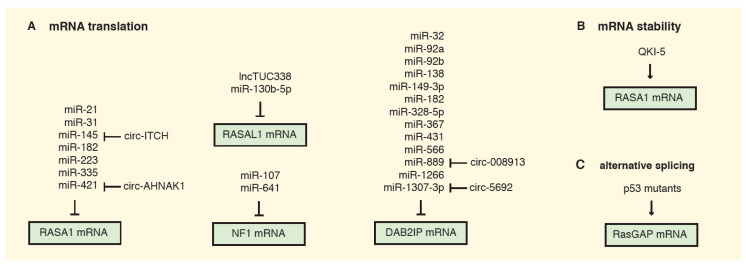
Schematic overview of current evidence for post-transcriptional regulation of RasGAPs. (**A**) MicroRNA-dependent regulation. Both miRNAs and long non-coding RNAs (lncRNA and circ-RNA) are involved in modulating RasGAP mRNA translation both directly and indirectly. (**B**) Control of mRNA stability. Stability of RASA1 mRNA can be fostered by direct binding with the RNA-binding protein Quaking-5 (QKI-5); loss of QKI-5 can reduce RASA1 expression in cancer. (**C**) Control of alternative splicing. Expression of missense p53 mutant proteins enhances the inclusion of cytosine-rich exons in the mRNA of RasGAPs; the encoded proteins show defects in membrane association and reduced Ras inhibitory function.

Non-coding RNAs compose almost 60% of the transcriptional output in human cells and create a complex regulatory network by controlling the mRNAs of multiple genes [72]. Not surprisingly, most RasGAPs can be regulated by ncRNAs (Figure 2).

One well-described target for regulation by ncRNAs is RASA1. By downregulating RASA1, miR-31, miR-21, miR-223, and miR-335 foster increased Ras activation and colon cancer cell proliferation, counteracting apoptosis [15,16,73,74]. Intriguingly, miR-31 is a transcriptional target for mutated K-RAS in pancreatic tumors, where miR-31-mediated RASA1 inhibition creates a positive feedback that facilitates RhoA activation and cell migration and invasion [75]. Similarly, the miR-21/RASA1 axis was described in cervical cancer and esophageal squamous cell carcinoma, where miR-21 enhances Ras signaling and EMT [19,76]. High levels of miR-21 were found in the serum of cervical cancer patients, suggesting that secreted miR-21 can mediate RASA1 inhibition in a cell non-autonomous manner [76].

The hypoxia-inducible miR-182 was reported to target two different RasGAPs: RASA1 in hepatocellular cancer and oral cavity squamous cells carcinoma, and DAB2IP in colorectal carcinoma [77,78,79]. Interestingly, miR-182-mediated RASA1 inactivation could also promote tumor vascularization by reprogramming the secretome of cancer cells: in fact, HUVECs (human umbilical vein endothelial cells) treated with culture medium from RASA1-depleted cells showed increased capillary-like structure formation [77].

In gastric cancer, the upregulation of miR-107 and miR-130b-5p was shown to promote proliferation, migration, and invasion of cancer cells, partially via reduction of their respective targets NF1 and RASAL1 [80,81].

RasGAP downregulation can also contribute to chemoresistance. Non-small cell lung cancer cells can develop resistance to EGFR tyrosine kinase inhibitor (TKI)-mediated therapy by upregulating miR-641. This miRNA targets NF1 and enhances Ras/MEK/ERK signaling, and allows cells to bypass EGFR TKI treatment [82]. Similarly, upregulation of miR-32 downregulates DAB2IP in prostate cancer, inducing autophagy via mTOR pathway activation and counteracting radiotherapy-induced apoptosis, thus leading to increased cancer cell survival after ionizing radiation treatment [83].

RasGAPs can also be regulated by circular RNAs (circRNA), transcripts that are frequently downregulated in cancer, and often function as tumor suppressors by competitive binding with other ncRNAs (largely miRNAs). For instance, low expression of circ-ITCH combined with increased expression of the RASA1-targeting miR-145 predicts poor prognosis in ovarian cancer. The circ-ITCH can act as competing endogenous RNA (ceRNA) to sponge miR-145 and enhance RASA1 expression and inhibit malignant progression of ovarian cancer [84]. Similarly, circ-AHNAK1 may enhance RASA1 expression in TNBC through sponging miR-421 [18].

Conversely, the long non-coding RNA TUC338, which is highly expressed in hepatocellular carcinoma, downregulates RASAL1 expression through an unclear mechanism, thereby stimulating Ras signaling [85].

A number of miRNAs have been reported to target DAB2IP, facilitated by its long 3′ UTR sequence. Since DAB2IP modulates numerous oncogenic pathways, miRNA-mediated DAB2IP inhibition can potentially have broad effects on cell fate [30,86]. For instance, miR-889 inhibits DAB2IP expression in esophageal squamous cell carcinoma and colorectal cancer [87,88]. Intriguingly, miR-889 is upregulated upon arsenite exposure via repression of its competitive circRNA-008913: the consequent DAB2IP downregulation and acquisition of cancer stem cell properties could partially explain the carcinogenic effect of arsenite [89]. A similar regulatory loop has been described in hepatocellular carcinoma, where DAB2IP mRNA and protein levels were upregulated upon overexpression of circ-5692, able to sponge the DAB2IP-targeting miR-328-5p [90]. Several miRNAs of the miR-92a family were reported to target DAB2IP in multiple tumors [86,91,92,93]. DAB2IP inhibition by miR-556 in bladder and esophageal cancer fosters cells proliferation and sustains tumor progression via aberrant Ras-MAPK signaling activation [94,95]. Intriguingly, miR-556 was detected in the plasma of bladder cancer patients, suggesting that this miRNA could repress DAB2IP in a cell non-autonomous manner. Analogously, a cell non-autonomous effect was described by us for miR-149-3p in prostate cancer; this miRNA is secreted by cancer cells and can reduce DAB2IP levels in endothelial cells (ECs), potentially remodeling the tumor microenvironment [86]. Various additional miRNAs have been described to target DAB2IP in different cancers [96,97,98,99,100], and it is reasonable to predict that more will be discover in the future.

The literature on miRNA-dependent inhibition of RasGAPs is currently limited to a few family members. However, a quick search using public prediction tools revealed that a consistent number of cancer-related miRNAs have the potential to target one or more human RasGAPs (Table 2). It is likely that complex post-transcriptional regulatory networks can modulate expression of RasGAPs in cancer, affecting the amplitude and duration of Ras activity, with implications for the cell’s response to extracellular inputs. Further investigation in this area is needed.

In addition to miRNAs, other mechanisms can affect RasGAPs mRNA stability and translation. For instance, RASA1 levels are controlled by the RNA-binding protein Quaking (QKI)-5 (Figure 2B). QKIs are a conserved family of proteins that promote stability and translation of mRNAs. QKI-5 binds human RASA1 within its 3′ UTR, enhancing its translation and thus sustaining its GAP activity. In kidney tumorigenesis, loss of QKI-5 destabilizes RASA1 mRNA and favors Ras signaling-mediated cancer cell survival and tumor growth [104].

Finally, a recent study uncovered a mechanism by which the splicing of GAPs can be reprogrammed to foster Ras activation in pancreatic ductal adenocarcinoma (PDAC) [105]. Specifically, expression of oncogenic p53 mutant proteins (mutp53) promotes the inclusion of cytosine-rich exons within the mRNA of multiple GAPs, resulting in defective Ras inhibition. The alternatively spliced GAPs apparently retain their GTPase-activating function, but are defective in membrane association and binding to active Ras (Figure 2C). Authors have clearly described this phenomenon for ARHGAP17, a Rho GTPase documented to counteract Rac1 and Ras signaling [106], but also explicitly suggest that it could apply to other GAPs. PDAC is frequently driven by co-existing mutation of K-RAS and TP53, but the biological impact of their cooperation is not fully understood. Interestingly, some mutant K-Ras proteins remain susceptible to GAP-mediated GTP hydrolysis, so RasGAPs can buffer the oncogenic potential of RAS mutation [51,107]. By regulating the alternative splicing of GAPs, mutp53 would contribute to maximal activity of mutant K-Ras, enhancing its oncogenic potential. This model was supported by experiments in PDAC preclinical models [105].

## 6. Post-Translational Inhibition of RasGAPs in Cancer

### 6.1. Phosphorylation

Little is known about post-translational modifications of RasGAPs, but the activity of some of these proteins is regulated by phosphorylation.

RASA1 undergoes receptor-mediated phosphorylation on Tyr-460, between the SH2 and the PH domains, and this modification possibly affects its interaction with signaling proteins at the membrane [108].

**Figure 3:**
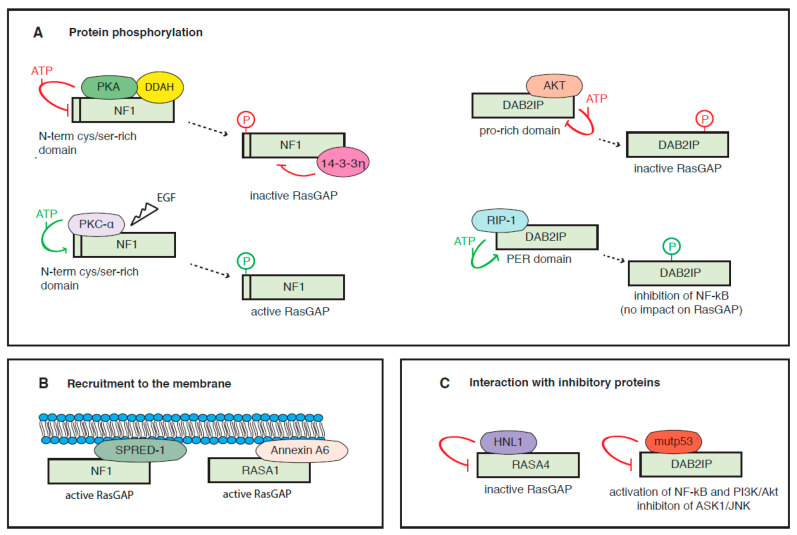

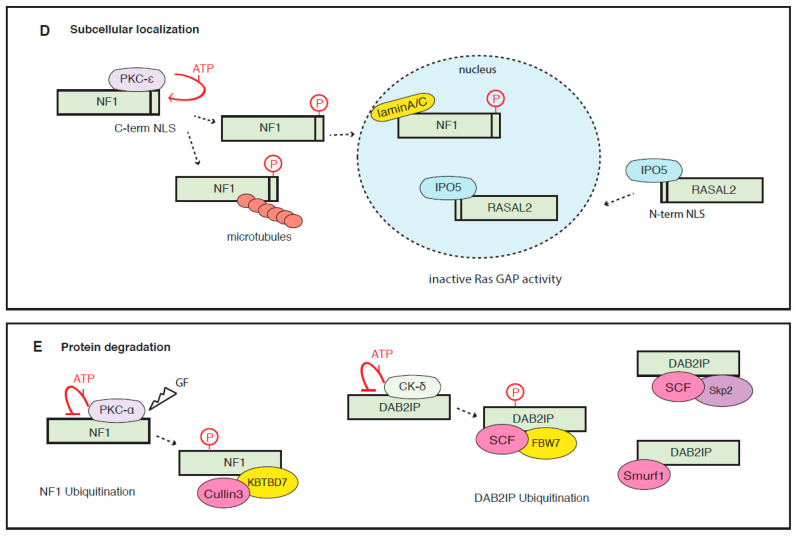
Post-translational regulation of RasGAPs. Examples are shown of RasGAPs that are regulated by protein modification, cellular localization, interaction with other proteins, and degradation via proteasome. (**A**) The activity and stability of some RasGAPs can be regulated by phosphorylation. NF1 is inhibited by protein kinase A (PKA)-mediated phosphorylation at the N-terminus, marking the protein for binding with 14-3-3η. NF1 also undergoes activating phosphorylation by protein kinase C-alpha (PKC-α) in cells treated with epidermal growth factor (EGF). DAB2IP can be inhibited by Akt-mediated phosphorylation on a proline-rich domain or stimulated by receptor interacting protein-1 (RIP-1)-mediated phosphorylation on the PER domain. (**B**) The activity of RasGAPs is enhanced by membrane recruitment, increasing local protein concentration and facilitating interaction with active Ras. NF1 interaction with SPRED-1 (sprout related EVH1 domain containing 1), and RASA1 interaction with Annexin A6 stimulate their membrane localization and Ras inhibitory activity. RasGAPs can also be recruited to the membrane by interaction with specific lipids, including phosphatidic acid, phosphatidylinositol phosphates, arachidonic acid, and eicosanoids (not shown). (**C**) The binding with interacting proteins can affect RasGAP cellular localization and activity. HNL1 can bind RASA4, and missense mutant p53 proteins can bind DAB2IP, interfering with their ability to bind their physiological targets. (**D**) RasGAPs can be sequestered in the nucleus, reducing their functional interaction with active Ras. When phosphorylated in the C-terminal domain, NF1 traslocates to the nucleus, where it interacts with lamin A/C. NF1 can also be delocalized by binding to tubulin (see text for more detail). RASAL2 is a cargo for importin 5 (IPO5). (**E**) Some RasGAPs undergo proteasomal degradation by specific ubiquitin-ligases. NF1 is polyubiquitinated upon PKC-mediated phosphorylation by a Cullin3/KBTBD7 (kelch repeat and BTB domain containing 7) complex. DAB2IP can be ubiquitinated in response to different conditions by at least three different E3: the FBW7 (F-box and WD repeat domain-containing 7)/SCF (skip1-Cul1-F-box protein) complex, the Skp2 (S-phase kinase-associated protein2)/SCF complex, and Smurf1 (SMAD-specific E3 ubiquitin-ligase 1).

NF1 can be phosphorylated by protein kinase A (PKA) on a cysteine/serine-rich domain in the N-terminal region of the protein (Figure 3A). This modification favors the inhibitory binding with 14-3-3η proteins, thus negatively regulating its GAP activity [109,110]. In an apparent contradiction, another study reported that protein kinase C-alpha (PKC-α) phosphorylation of NF1 on the cysteine/serine-rich domain is essential to sustain RasGAP activity in neural cells treated with epidermal growth factor (EGF) [111]. Adding further complexity, it has been reported that PKC-α phosphorylates NF1 in response to growth factors, addressing this protein for proteasomal degradation in murine embryonic fibroblasts and glioblastoma cells [112,113]. Besides that, NF1 can also be phosphorylated in the C-terminal region, affecting its nuclear localization (see below).

DAB2IP can be phosphorylated by Akt on a serine residue within a proline-rich domain in the C-terminal region of the protein, inhibiting its functions: phosphorylated DAB2IP shows reduced interaction with Ras and TRAF2 (TNF-receptor associated factor 2), facilitating Ras signaling and preventing activation of the pro-apoptotic ASK1 (apoptosis signal-regulating kinase 1) pathway [114]. On the other hand, a stimulatory phosphorylation of DAB2IP in the central region of the protein is carried out by RIP-1 kinase [115]. However, this last modification is not necessary for the RasGAP activity of DAB2IP; in fact, overexpression of a non-phosphorylatable DAB2IP mutant could not suppress NF-κB signaling, but was still able to dim Ras activation [66,115].

Finally, a recent study uncovered that RASAL2 can be phosphorylated on Serine 237 within the PH domain. The impact on Ras has not been analyzed, but increased phospho-RASAL2 was detected in aggressive ER (estrogen receptor) negative breast cancer cells, leading authors to propose that this modification could shift the tumor-suppressive function of the protein to a tumor-promoting activity, possibly explaining some contradictory observations on its role in cancer [29].

In addition to the above specific examples, it appears that most RasGAPs are subject to phosphorylation and other post-translational modifications; in fact, a number of high-confidence modification sites have been assigned to the various RasGAPs using proteomic discovery mass-spectrometry (PhosphoSitePlus database, https://www.phosphosite.org/) [116]. It remains to be established how these modifications can affect RasGAP functions, and whether they occur differentially in normal versus cancer cells. Further research in this direction may potentially uncover novel promising drug targets.

### 6.2. Protein–Protein Interactions

Formation of complexes with other molecules can modulate the activity of RasGAPs, favoring or hampering their function (Figure 3B,C).

One such example is the signaling protein SPRED-1 (sprout related EVH1 domain containing 1), whose inactivation causes Legius disease, a rare genetic skin pigmentation disorder that resembles Neurofibromatosis Type 1 [117]. Notably, SPRED-1 promotes the recruitment of NF1 to the plasma membrane, where it can dampen growth factor-induced Ras activation. This observation also partially explains the overlapping pathophysiology of NF1 and Legius syndromes [117]. Mechanistically, SPRED-1 and K-Ras bind NF1 via two different interfaces, forming a ternary complex. The structural basis of this interaction has been recently detailed in a study that also found that SPRED-1 phosphorylation on Ser 105 can disrupt the SPRED-1/NF1 interaction [118]. Notably, SPRED-1 can be phosphorylated by oncogenic activation of EGFR (L858R) in lung adenocarcinoma cells, thus indirectly facilitating Ras signaling activation [118].

Analogously, the adaptor protein Annexin A6 is a positive modulator of GAP activity; in fact, it facilitates RASA1 localization to the cell membrane, promoting its inhibitory action on activated Ras [119].

In contrast, the protein Src Homology Phosphatase 2 (SHP2) enhances the duration and intensity of Ras signaling by specifically dephosphorylating an autophosphorylated tyrosine that provides a docking site for the SH2 domain of RASA1 (p120RasGAP) on activated HER2 and EGFR receptors [120]. Accordingly, inhibition of SHP2 binding using a substrate-derived phosphopeptide significantly reduced mitogenic and survival signaling in HER2+ breast cancer cells [121].

Another inhibitor, the enzyme dymethylarginine dimethylaminohydrolase (DDAH), a known NO/NOS cellular regulator, binds NF1 in the C-terminal domain, favoring its interaction with PKA and consequent inhibitory phosphorylation, thus resulting in reduced RasGAP activity [122].

The protein encoded by the HN1L gene is overexpressed in NSCLC and correlates with enhanced tumor growth and poor prognosis. HN1L can bind RASA4, and this interaction reduces its GTPase activity, thereby fostering Ras-MAPK signaling and promoting cell proliferation, without affecting apoptosis or cell senescence [123].

Finally, tumor-associated missense mutant p53 proteins can bind DAB2IP, interfering with its functions (Figure 3C). Association with mutp53 prevents DAB2IP interaction with the kinase ASK1; as a consequence, mutp53 inhibits the pro-apoptotic ASK1/c-Jun N-terminal kinase (JNK) axis, while enhancing oncogenic NF-κB activity in response to inflammatory signals [124]. Similarly, mutp53 prevents DAB2IP interaction with Akt, enhancing activation of the pro-survival PI3K/Akt pathway in response to insulin [125]. On these grounds, it is reasonable to anticipate that mutp53 can also prevent DAB2IP interaction with Ras, enhancing MAPK signaling in cancer cells; however, this hypothesis awaits experimental validation.

### 6.3. Subcellular Localization

Localization at the membrane is important for RasGAPs function and can be altered in cancer cells, resulting in aberrant Ras activation (Figure 3D).

NF1 can be phosphorylated by protein kinase C-epsilon (PKC-ε) in the C-terminal region, and this modification triggers nuclear import of NF1 and its binding with lamin A/C in the nuclear envelope. Nuclear localization is cell-cycle regulated, peaking in the G2 phase; in mitosis, NF1 localizes on the centrosome and mitotic spindle, contributing to chromosome congression [126]. Binding with tubulin or lamin A/C alters cell localization of NF1, affecting its RasGAP function. Accordingly, when associated with tubulin, NF1 showed a four-fold reduction in affinity to N-Ras and was less active in stimulating its GTPase activity [127]. Recent evidence indicates that NF1 is a transcriptional co-repressor of Estrogen Receptor (ER) alpha in mammary epithelial cells, thus revealing an additional GAP-independent tumor-suppressive function linked to its nuclear localization. Accordingly, NF1 loss correlates with tamoxifen resistance in ER+ breast cancers, and co-targeting of MEK and ER may improve treatment of NF1-deficient tumors [128].

Similarly, RASAL2 is a cargo for the importin IPO5 in colorectal cancer. IPO5 sequesters RASAL2 by binding an NLS located in the N-terminal region, and prevents RASAL2 function as a signaling pathway suppressor [129].

### 6.4. Protein Degradation

There is also evidence of RasGAP proteins destabilization in cancer (Figure 3E). For instance, stimulation with growth factors can rapidly induce proteasomal degradation of NF1. Phosphorylation-dependent ubiquitination of NF1 is facilitated by the scaffold protein Cullin3, complexed with the adaptor protein KBTBD7 (BTB domain-containing kelch-repeat and BTB domain containing 7). Formation of this complex dictates both the duration and the amplitude of Ras activation [112]. In glioblastoma, this chain of events fostering Ras signaling is sufficient for cell transformation and tumorigenesis [112,113]. At least three ubiquitin-ligases have been reported to mediate DAB2IP turnover, thus fostering Ras activation. One is Fbw7 (F-box and WD repeat domain-containing 7), complexed with the Cullin-Ring based E3 ligase SCF (skip1-Cul1-F-box protein). DAB2IP contains two potential phospho-degron sequences, with homology to consensus Fbw7 substrates, and degradation of DAB2IP by Fbw7/SCF in cancer cell lines possibly requires CKδ-mediated phosphorylation [114]. The second is the Skp2 (S-phase kinase-associated protein 2) /SCF complex in prostate cancer. Notably, DAB2IP can downregulate Skp2 expression, likely via Akt inhibition, while Skp2-mediated DAB2IP turnover is stimulated by Akt, thus establishing a feedback regulatory circuit [130]. The third is the Nedd4-like E3 ligase Smurf1 (SMAD-specific E3 ubiquitin-ligase 1) in ovarian cancer. Similarly to Skp2, Smurf1 is also stabilized by Akt-mediated phosphorylation; thus aberrant Akt activation can reduce DAB2IP protein levels by fostering the activity of at least two distinct ubiquitin-ligases [131,132].

## 7. Control of RasGAPs Levels and Functions by Extracellular Inputs

**Figure 4:**
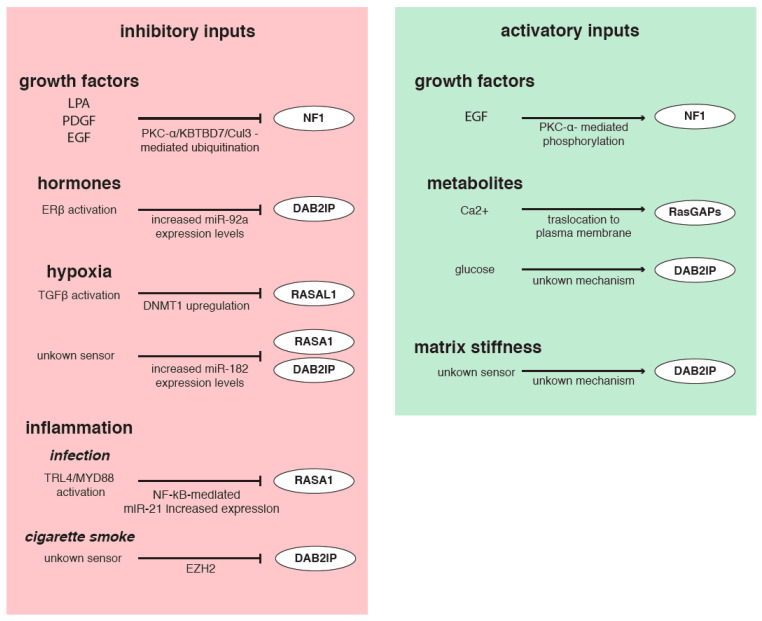
Multiple extracellular inputs can affect intracellular RasGAPs levels and activities in cancer cells. Schematic representation of extracellular signals that inhibit (red) or stimulate (green) expression and/or activity of RasGAPs, thus potentially affecting Ras signaling and oncogenic cell behaviors (see text for details).

As we have seen, various molecular mechanisms are involved in regulating RasGAPs, thus indirectly controlling Ras activity. Notably, alterations of RasGAPs transcription, translation, and function can occur in response to extracellular inputs, including changes in the microenvironment, mechanical cues, and a variety of signaling molecules (Figure 4).

Growth factors are constantly released in the TME by stromal and cancer cells and strongly impact on tumor progression [133]. Exposure to lysophosphatidic acid (LPA), platelet derived growth factor (PDGF), and EGF was shown to promote NF1 phosphorylation via PKC-α and its ubiquitination and rapid destruction in mouse embryonic fibroblasts and glioblastoma cells [112,113,134]. However, Mangoura et al. conversely described a PKC-α activatory phosphorylation of NF1 promoted by EGF stimulation [111]. It is possible that not all growth factors trigger NF1 degradation, or that in some cell types this inhibitory mechanism is not functional [113,134].

Hormone receptor levels and functions can also affect RasGAPs. In bladder cancer, estrogen receptor β (ERβ) increases transcription of miR-92a, consequently decreasing DAB2IP levels and promoting cancer growth and invasion [93]. Importantly, aberrant expression of ERα/β is associated with a variety of cancers, suggesting that ERβ-dependent regulation of DAB2IP might be implicated in other tumor types [135].

Various inflammatory inputs can regulate RasGAPs via direct and indirect mechanisms. For example, the inflammatory response induced in colorectal cancer cell lines upon *Fusobacterium nucleatum* infection triggers activation of the TLR4/MYD88/NF-κB axis that induces expression of miR-21, which inhibits RASA1 protein synthesis, fostering Ras activation and cell growth and proliferation [136]. Other miRNAs targeting RasGAPs are under the control of inflammatory stimuli, suggesting an indirect way to promote Ras signaling in response to inflammation. For example, NF-κB stimulates expression of miR-223, a RASA1-targeting miRNA [16], via binding its promoter [137]. Similarly, transcription of the miR-149 gene, encoding miRNAs targeting DAB2IP [86], can be stimulated or counteracted, respectively, by fibroblast growth factor 2 (FGF2) or tumor necrosis factor- alpha (TNF-α) [138,139].

Chronic inflammation linked to cigarette smoke is a common risk factor for pulmonary disorders, including Chronic Obstructive Pulmonary Disease (COPD) and lung cancer. Interestingly, cigarette smoke and consequent chronic inflammation of the airways were shown to induce epigenetic silencing of DAB2IP via EZH2. This phenomenon can favor uncontrolled epithelial cell proliferation, possibly prompting the progression of inflammatory diseases of the airways towards lung cancer [140].

Most GAPs have one or more C2 domains, structural modules that can bind calcium ions (Ca^2+^) and mediate interaction with phospholipids. Therefore, extracellular inputs that trigger dynamic changes in cytosolic Ca^2+^ concentration can potentially modulate RasGAP functions. At least RASA4 and RASAL1 are known to be regulated by Ca^2+^; intracellular mobilization of Ca^2+^ drives a rapid C2 domain-dependent translocation of these two proteins to the plasma membrane, increasing RasGAP activity [141,142]. Interestingly, RASA4 is also a GAP for Rap1, and changes specificity by forming monomers (functional as RasGAP) or homodimers (functional as Rap1 GAP) via a calcium-regulated process; consequently, Ca^2+^ levels can also coordinate the activation of Ras and Rap1 signaling pathways [143].

There is also evidence that environmental metabolites can regulate RasGAPs, with implications for cancer. For example, glucose shortage in the tumor niche is an unfavorable condition experienced by cancer and stromal cells, which leads them to reprogram their metabolism. Intriguingly, DAB2IP expression may be sensitive to extracellular glucose concentration: in endothelial cells grown in low glucose, mRNA and protein levels of DAB2IP are reduced if compared with high glucose, leading to HIF1-α (hypoxia inducible factor-alpha) activation and induction of VEGF (vascular endothelial growth factor) pro-angiogenic factor. The mechanism involved in glucose-dependent regulation of DAB2IP remains unknown [144].

Another common condition observed in the core of solid tumors is hypoxia, and there is evidence that low oxygen concentration can stimulate Ras activity by interfering with GAPs. For instance, hypoxia-activated TGF-β1 can induce hypermethylation of the RASAL1 promoter via upregulation of DNMT1 [63]. Furthermore, hypoxia stimulates production of miR-182, which is able to target both RASA1 and DAB2IP [77]. Notably, hypoxic stress reprograms the expression of multiple miRNAs via activation of HIF1-α, and several additional RasGAP-targeting miRNAs, such as miR-107, miR-130, miR-145, and miR-335, are upregulated by hypoxic conditions, potentially favoring Ras activation and tumor progression [145].

Finally, interaction with the extracellular matrix (ECM) affects the cytoskeleton and activates mechanosensory pathways that regulate crucial cell behaviors such as proliferation, EMT, chemoresistance, and self-renewal of cancer stem cells (CSCs). There is some evidence that changes in density and composition of the ECM, as well as mechanical stimuli from neighboring cells, can potentially modulate RasGAPs. For instance, the SH2 domain of RASA1 binds the phosphorylated, activated form of Focal Adhesion Kinase (FAK); this interaction dampens its GAP activity, enhancing Ras signaling. Similarly, NF1 binds microtubules via its GAP domain, and this interferes with its Ras inhibitory function. These interactions suggest a possible link between the cytoskeleton and Ras activity [127,146,147]. Finally, a recent study using human colon carcinoma cells showed that growth in a soft matrix significantly reduced DAB2IP protein levels, as compared to a stiffer substrate, thus promoting proliferation and self-renewal of colon cancer stem cells [148]. The molecular mechanism involved has not been elucidated, but this piece of evidence supports the concept that mechanical cues from the ECM can potentially affect Ras activity via modulation of RasGAP proteins. It is likely that more examples of such regulation will emerge in the future.

## 8. Conclusions

The frequency at which expression or function of RasGAPs is altered in cancer clearly highlights their importance as regulators of tissue homeostasis and suppressors of transformation. In some cases, loss of a single RasGAP can be enough to prompt tumor development, as in Neurofibromatosis Type 1. Alternatively, loss of a RasGAP may not be sufficient to induce cell transformation, but can amplify oncogenic signaling pathways, favoring tumor progression in response to aberrant environmental inputs, as probably occurs with DAB2IP and other GAPs.

Despite their importance, our comprehension of the mechanisms controlling the expression and activity of RasGAPs remains incomplete. In particular, we do not fully understand the possible functional redundancy of these genes, and we do not know if concomitant loss of two or more RasGAPs has a synergic effect resulting in fully uncontrolled Ras activation and a more aggressive tumoral phenotype. From this perspective, it would be interesting to study those miRNAs that could simultaneously target multiple RasGAPs (see Table 2). Furthermore, the current literature exploring RasGAPs inactivation in cancer is largely focused on a subset of these genes, but it is likely that many of the mechanisms we have reviewed here, and their biological consequences, can be extended to other family members.

Another crucial point is the role of extracellular inputs that negatively regulate RasGAPs. It is reasonable to assume that cancer-related signals in the tumor microenvironment (TME) can modulate RasGAPs activity in both cancer and non-cancer cells. Within a tumor, cancer cells may induce RasGAPs dysfunction in stromal cells by secreting growth factors and cytokines, but also by altering environmental conditions such as oxygen levels, availability of specific metabolites, or stiffness of the extracellular matrix. We are convinced that loss of function of RasGAPs in cells that infiltrate the tumor environment, such as endothelial cells and fibroblasts, can have a dramatic impact on tumor development, as hinted by studies on DAB2IP and RASAL3 [32,86]. From this perspective, signals released by cancer cells may indirectly amplify Ras-dependent responses in microenvironmental cells, fostering tumor aggressiveness; such signals may become attractive targets for combined therapy against malignancy progression.

## Figures and Tables

**Table 1:** Evidence of promoter methylation of RasGAPs in cancer.

Gene	Tumor	References
NF1	Hepatocellular carcinoma	[9]
RASA4	Myelomonocytic leukemia	[23]
RASAL1	Hepatocellular and nasopharyngeal carcinoma; breast, thyroid, and bladder cancer; NK/T-cell lymphoma	[9,25,26,63,68], reviewed in [69]
RASAL2	Renal carcinoma, breast cancer	[60,70]
RASAL3	Prostate CAFs	[32]
DAB2IP	Hepatocellular carcinoma; lung, breast, prostate, and gastrointestinal cancer; ovarian carcinoma	[9,67], reviewed in [30]

**Table 2:** Cancer-related miRNAs potentially targeting 10 human RasGAPs.

**(a) number of cancer-related miRNAs predicted to bind a single RasGAP**		
**RasGAP**	**TargetScan conserved sites**	**DIANA microT-CDS**
RASA1	42 (53)	111 (350)
NF1	33 (42)	150 (388)
RASA2	51 (57)	102 (288)
RASA3	0 (0)	15 (39)
RASA4	0 (0)	24 (121)
RASAL1	11 (11)	13 (34)
RASAL2	57 (79)	91 (354)
RASAL3	0 (0)	15 (58)
SYNGAP1	24 (34)	56 (257)
DAB2IP	31 (41)	41 (132)
**(b) cancer-related miRNAs predicted to bind at least four RasGAPs**		
**Targeted RasGAPs**		**onco-miRNAs**
NF1, RASA1, RASA2, RASA3, RASAL1, RASAL2, SYNGAP1		hsa-miR-3163
DAB2IP, NF1, RASA1, RASA2, RASA3, RASAL2		hsa-miR-548c-3p
DAB2IP, NF1, RASA1, RASA2, RASAL1, RASAL2		hsa-miR-5692a
NF1, RASA1, RASA2, RASA4, RASAL2		hsa-miR-5590-3p
DAB2IP, NF1, RASA1, RASA2, RASAL2		hsa-miR-582-5p
DAB2IP, NF1, RASA2, RASA3, RASAL2		hsa-miR-27b-3p, hsa-miR-27a-3p
NF1, RASA2, RASAL1, RASAL2, SYNGAP1		hsa-miR-4282
NF1, RASA1, RASA2, RASA4		hsa-miR-129-5p
NF1, RASA1, RASA2, RASAL2		hsa-miR-30d-5p, hsa-miR-30b-5p, hsa-miR-320c, hsa-miR-4429, hsa-miR-30a-5p, hsa-miR-30e-5p, hsa-miR-320b, hsa-miR-495-3p, hsa-miR-33a-3p, hsa-miR-320d
NF1, RASA1, RASA2, SYNGAP1		hsa-miR-224-3p
DAB2IP, NF1, RASA,1, RASA3		hsa-miR-588
NF1, RASA2, RASA3, RASAL2		hsa-miR-543
NF1, RASA2, RASA4, RASAL2		hsa-miR-4775
DAB2IP, NF1, RASA2, RASAL2		hsa-miR-126-5p
DAB2IP, NF1, RASA2, SYNGAP1		hsa-miR-130a-5p
DAB2IP, RASA4, RASAL1, RASAL3		hsa-miR-1285-3p
RASA4, RASAL2, RASAL3, SYNGAP1		hsa-miR-1275

(**a**) miRNAs targeting each of the 10 RasGAPs were predicted using the TargetScan algorithm v.2.7 (http://www.targetscan.org/) or the DIANA microT algorithm v.5 (http://diana.imis.athena-innovation.gr/DianaTools/) and intersected with the list of cancer-related microRNAs from the miRcancer database (June 2020 release; http://mircancer.ecu.edu/). Numbers in parentheses indicate the total number of miRNAs, including those not included in the miRcancer database, predicted by each platform [101,102,103]. (**b**) Cancer-related miRNAs predicted by the DIANA microT algorithm were intersected to identify those targeting at least four different RasGAPs.
